# Taxonomic and Metagenomic Analyses Define the Development of the Microbiota in the Chick

**DOI:** 10.1128/mbio.02444-22

**Published:** 2022-12-08

**Authors:** Lydia Bogomolnaya, Marissa Talamantes, Joana Rocha, Aravindh Nagarajan, Wenhan Zhu, Luisella Spiga, Maria G. Winter, Kranti Konganti, L. Garry Adams, Sebastian Winter, Helene Andrews-Polymenis

**Affiliations:** a Department of Microbial Pathogenesis and Immunology, Texas A&M University, College Station, Texas, USA; b Deparment of Biomedical Sciences, Marshall University, Huntington, West Virginia, USA; c Interdisciplinary Program in Genetics, Texas A&M University, College Station, Texas, USA; d Department of Microbiology and Immunology, UT Southwestern Medical Center, Dallas, Texas, USA; e Department of Pathology, Microbiology and Immunology, Vanderbilt University, Nashville, Tennessee, USA; f Department of Veterinary Pathobiology, College of Veterinary Medicine, Texas A&M, College Station, Texas, USA; University of Michigan—Ann Arbor

**Keywords:** chickens, microbiota development, *Salmonella*, branched-chain amino acids, pathogen

## Abstract

Chicks are ideal to follow the development of the intestinal microbiota and to understand how a pathogen perturbs this developing population. Taxonomic/metagenomic analyses captured the development of the chick microbiota in unperturbed chicks and in chicks infected with Salmonella enterica serotype Typhimurium (STm) during development. Taxonomic analysis suggests that colonization by the chicken microbiota takes place in several waves. The cecal microbiota stabilizes at day 12 posthatch with prominent *Gammaproteobacteria* and *Clostridiales*. Introduction of *S.* Typhimurium at day 4 posthatch disrupted the expected waves of intestinal colonization. Taxonomic and metagenomic shotgun sequencing analyses allowed us to identify species present in uninfected chicks. Untargeted metabolomics suggested different metabolic activities in infected chick microbiota. This analysis and gas chromatography-mass spectrometry on ingesta confirmed that lactic acid in cecal content coincides with the stable presence of enterococci in STm-infected chicks. Unique metabolites, including 2-isopropylmalic acid, an intermediate in the biosynthesis of leucine, were present only in the cecal content of STm-infected chicks. The metagenomic data suggested that the microbiota in STm-infected chicks contained a higher abundance of genes, from STm itself, involved in branched-chain amino acid synthesis. We generated an *ilvC* deletion mutant (*STM3909*) encoding ketol-acid-reductoisomerase, a gene required for the production of l-isoleucine and l-valine. Δ*ilvC* mutants are disadvantaged for growth during competitive infection with the wild type. Providing the *ilvC* gene in *trans* restored the growth of the Δ*ilvC* mutant. Our integrative approach identified biochemical pathways used by STm to establish a colonization niche in the chick intestine during development.

## INTRODUCTION

Nontyphoidal Salmonella (NTS), Salmonella enterica subsp. *enterica* serovars Typhimurium and Enteritidis, are the leading cause of bacterial foodborne gastroenteritis in humans and livestock worldwide (1–3). Since the first report of food poisoning associated with NTS in 1888, infections in livestock and concurrent human cases of foodborne salmonellosis have increased. The overall rate of NTS infections has not declined in 50 years in the United States, and this problem is reflected around the world (4). The World Health Organization (WHO) estimates that *S.* Enteritidis and *S.* Typhimurium cause approximately 80% of all human cases, 94 million cases of gastroenteritis worldwide, and 155,000 deaths (3). Currently in the United States, NTS causes about 1.35 million infections, 26,500 hospitalizations, and 420 deaths annually resulting in $400 million in direct medical costs (5). There are currently no effective preventative or treatment strategies available for reducing NTS infection in humans.

NTS gastroenteritis in developed countries is primarily a foodborne infection that is frequently associated with contaminated chicken meat and eggs (3). Between 2004 and 2008, approximately 50% of NTS outbreaks were from poultry and eggs (29 and 18% respectively) (CDC National Outbreak Reporting System, 2004 to 2008). Control methods for NTS in chickens currently include eliminating vertical transmission in breeding stock and replacement chicks, reducing feed and environmental contamination, and improved biosecurity (1). Infected chickens are difficult to identify because they are subclinically colonized with Salmonella and show no clinical signs of infection (6). Broiler chicks contract NTS infection in the first few days posthatch and can develop lifelong subclinical infections (7, 8). These subclinical infections with NTS persist in >90% of birds at 8 to 9 weeks of age (well within the age at which commercial broilers are slaughtered) (8). The national prevalence of NTS contamination in chicken carcasses is approximately 31% (16.8% *S.* Enteritidis and 14.5% *S.* Typhimurium) (9).

Reduction in subclinical intestinal carriage of NTS in chickens is a key strategy for reducing human NTS gastroenteritis. Such strategies require understanding of the NTS mechanisms for colonization of the chick intestine to develop new methodologies. Newly hatched chicks have sterile gastrointestinal (GI) tracts and are highly susceptible to deadly NTS infection (<4 days posthatch) (10). In mammals, NTS cleverly exploits the intestinal environment and intestinal inflammation to outcompete the intestinal microbiota (11–14). In mice, NTS employs its type 3 secretion system 1 (TTSS-1) to promote a massive infiltration of neutrophils that in turn liberates reactive oxygen and nitrogen species (15). NTS encodes multiple oxidases/reductases for terminal electron acceptors and can use these nutrients under anaerobic conditions to catabolize a variety of fermentation products (1,2-propanediol, succinate, ethanolamine, and fructose-asparagine) produced in this inflammatory environment (16). When infected with *S*. Typhimurium at 4 days posthatch, chicks are robustly colonized in the intestinal tract but do not develop strong heterophilic inflammation (17). Thus, we expect that some of the mechanisms involved in NTS colonization of the developing chick intestine will be distinct from those of mammals. These new mechanisms will add to the already extensive repertoire of mechanisms utilized by Salmonella to dominate the intestinal lumen (17).

To further understand NTS requirements for colonization of the chick GI tract, we used taxonomic, metagenomic, and both untargeted and targeted metabolomic analyses of the microbiota during chick development over the first 19 days posthatch to capture the development of the microbiota to maturity. Untargeted metabolomics and metagenomics allowed us to identify significant pathways needed by Salmonella during colonization of the chick intestine, including branched-chain amino acid biosynthesis. Mutational analysis of *ilvC* (*STM3909*), ketol-acid-reductoisomerase, confirmed the necessity for biosynthesis of l-isoleucine and l-valine by STm during colonization of the chick intestine during early development. Thus, our integrative approach allowed us to identify biochemical pathways used by STm to colonize the developing chick gastrointestinal tract.

## RESULTS

### *Salmonella* infection in chicks does not affect weight gain or induce gross pathological changes.

**FIG 1:**
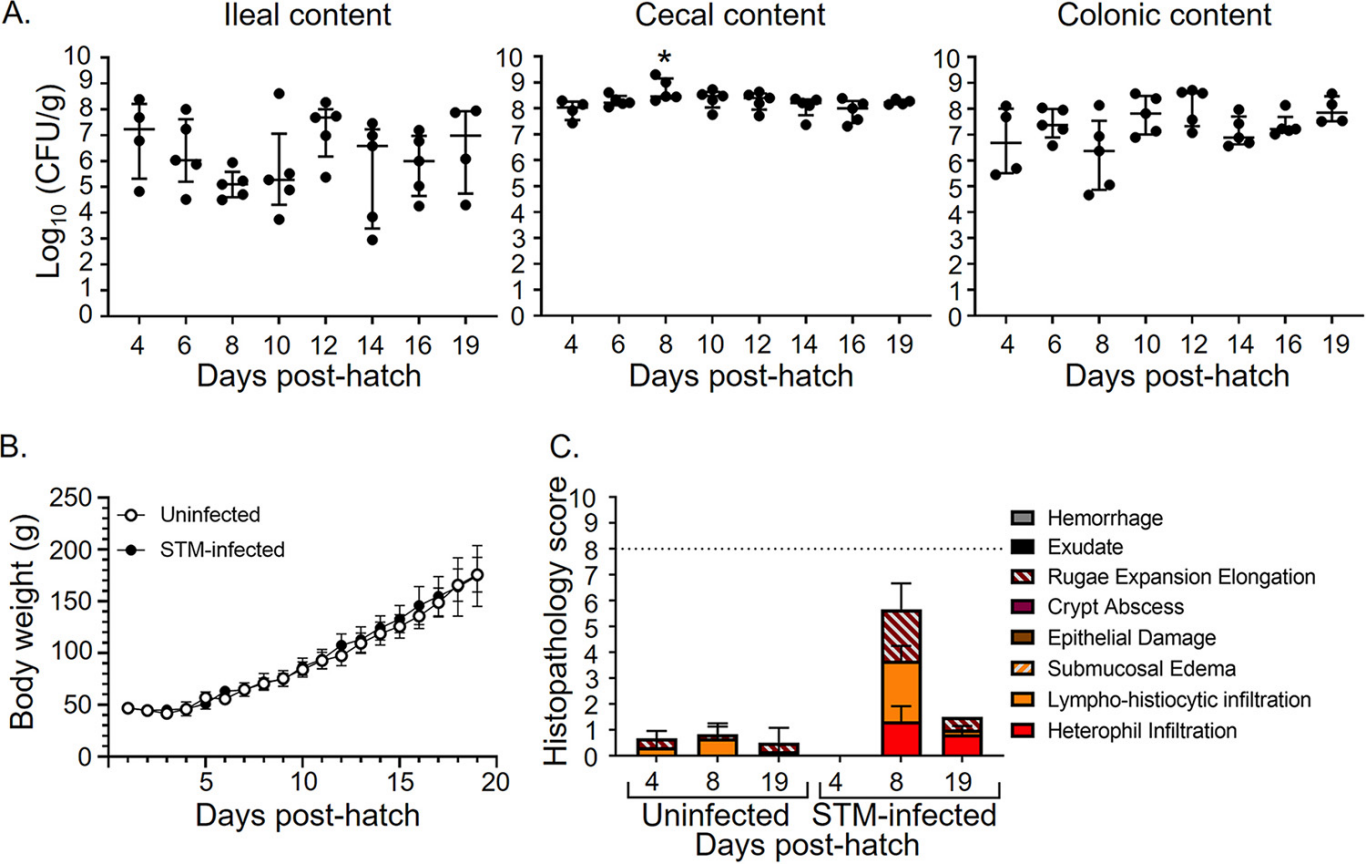
Salmonella Typhimurium infection results in prolonged colonization of the intestine without significant adverse affects on chicks. (A) Oral inoculation of 4-day-old chicks with 10^8^ CFU Salmonella enterica serotype Typhimurium ATCC 14028 spontaneously nalidixic acid derivative HA420 leads to stable colonization of the ileum, cecum, and colon. (B) Weight gain of chicks infected with STm compared to uninfected chicks. (C) Cecal sections were collected from STm-infected or uninfected chicks at days 4, 8, and 19 posthatch, fixed in formalin, paraffin embedded, cut, and stained with hematoxylin and eosin. Stained sections were scored for the signs of inflammation. A combined score of <8 corresponds to normal or mild inflammation; a combined score of >8 indicates moderate to severe inflammation.

We hatched chicks from specific-pathogen-free (SPF) eggs and divided them into two groups. One group was left unperturbed, while the other group was infected at 4 days posthatch with 10^8^ CFU of Salmonella enterica serotype Typhimurium ATCC 14028s. We monitored weight gain daily and STm colonization every 2 days until 19 days posthatch. Infected chicks became stably colonized with STm in the ileum, cecum, and colon within 5 h postinoculation and remained so for the duration of our study (Fig. 1A). STm-infected chicks and uninfected chicks gained weight indistinguishably (Fig. 1B). Furthermore, cecal tissue from days 4, 8, and 19 of age from either uninfected chicks or from chicks infected at 4 days of age suggests only mild damage to the intestinal epithelium after STm infection (Fig. 1C). This damage was apparent only on day 8 posthatch (day 4 postinfection) in STm-infected chicks and included mild heterophil infiltration, mild lympho-histiocytic infiltration, and mild expansion of the rugae. Interestingly, the observed damage coincided with a modest but statistically significant increase in cecal STm colonization at day 4 postinfection (Fig. 1A).

### Cecal microbiota develops in waves, and this development is disrupted by STm infection.

**FIG 2:**
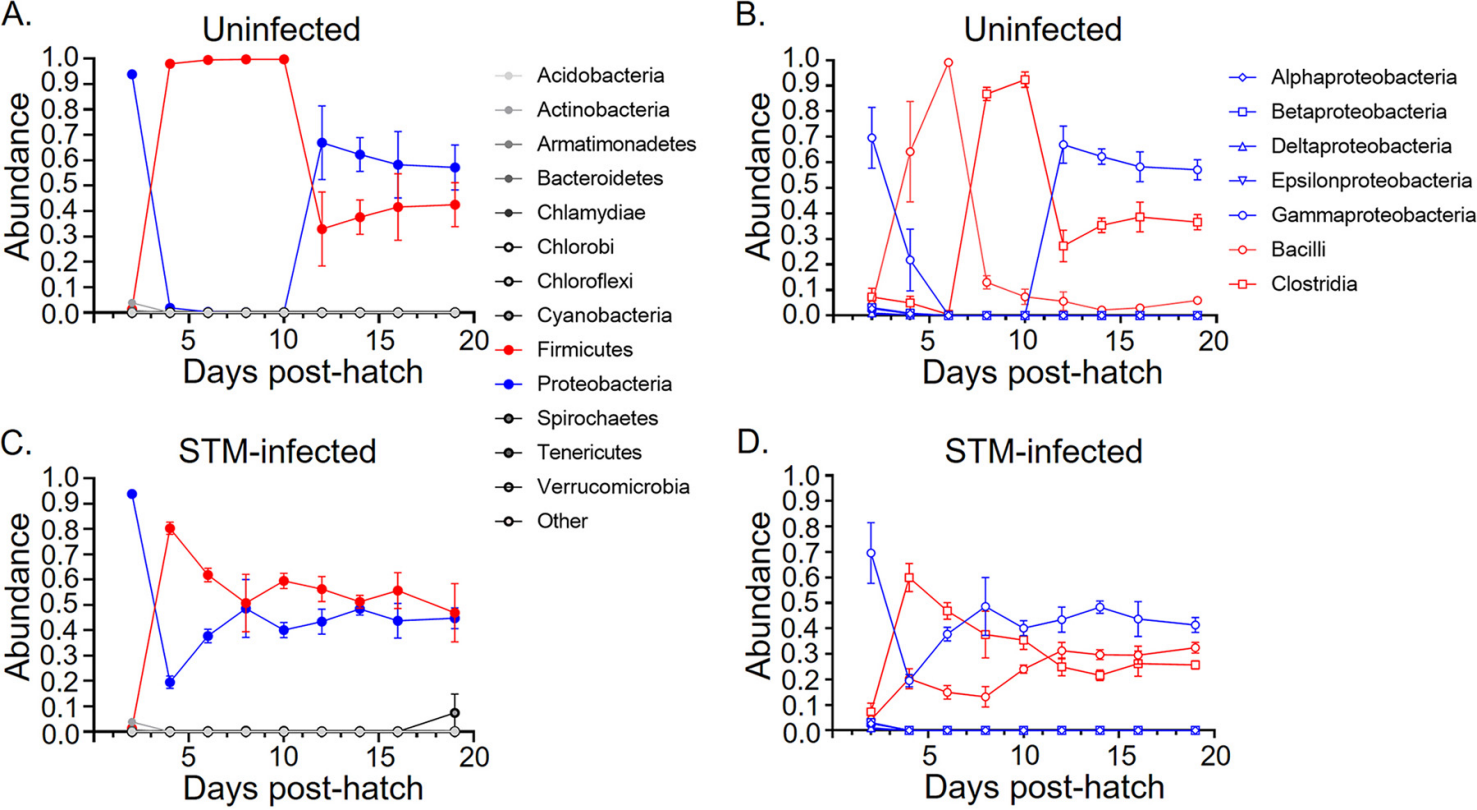
16S rRNA gene sequencing analysis of chicken cecal microbiota development over the first 19 days of life. (A and C) Composition of microbiota present in the ceca of uninfected (A) or STM-infected (C) chicks at the phylum level. (B and D) Composition of microbiota present in the ceca of uninfected (B) or STM-infected (D) chicks at the class level.

We sampled the cecal contents of chicks every other day from 2 days after hatching to 19 days of age to define the dynamics of development of the microbiota using 16S rRNA gene sequencing. Two phyla, *Firmicutes* and *Proteobacteria*, dominated the microbiota in developing chicks from hatching to the development of a stable microbiota by day 19 posthatch, both in unperturbed chicks (Fig. 2A) and in chicks infected with STm at day 4 posthatch (Fig. 2C). This finding is in contrast to the intestinal microbiota of mammals, which is more complex and is dominated by *Bacteroides* and *Clostridia* (18, 19). Stability and maturity in the composition of the cecal microbiota at the phylum level occurs between days 10 and 12 posthatch.

Looking at the microbiota colonizing the chick ceca at the class level, the ceca of unperturbed chicks are colonized in multiple waves, reaching a stable state by day 12 posthatch (Fig. 2B). Although sampling at day 2 posthatch is difficult and the cecal microbiota are sparse, Gammaproteobacteria were the most abundant member of the chick cecal population. The Gammaproteobacteria population diminished by day 6 posthatch, and Bacilli dominated the microbiota reaching a peak at 6 days postinfection. Between days 6 and 8 posthatch, Bacilli decreased in abundance, and Clostridia dominated the cecal population by day 10 posthatch. By day 12 posthatch, Gammaproteobacteria emerged again as approximately 60% of the cecal population, and were followed in prominence by the *Clostridiales* and to a lesser extent, Bacilli. After day 12 postinfection, the balance of Gammaproteobacteria, Clostridia and Bacilli appeared to be relatively stably maintained until the end of sampling at day 19 posthatch.

When chicks were orally infected with 10^8^ STm at 4 days posthatch, STm quickly altered the dynamics of the developing microbiota in the chick cecum (Fig. 2D). With the reduction in the relative abundance of the *Gammaproteobacteria*, the second wave of colonization is by *Clostridia*, which dominate the population by 8 days posthatch (day 4 postinfection). At that time, *Gammaproteobacteria* and *Bacilli* each represent about 20% of the cecal population, while the *Clostridia* are present in 40 to 60% relative abundance. By day 8 posthatch, the *Gammaproteobacteria* once again are dominant, not surprisingly because these are primarily STm, while the relative abundance of the *Clostridia* continues to decline. By day 12 posthatch, *Gammaproteobacteria* remain dominant but are closely followed in relative abundance by *Bacilli* and *Clostridia*. Thus, quickly after the introduction of STm into the intestinal tract of the developing chick, STm modulates the composition of the cecal microbiota and forms a stable community that differs from that in unperturbed chicks.

### Metagenomic shotgun sequencing identifies species present in developing chick microbiota.

**FIG 3:**
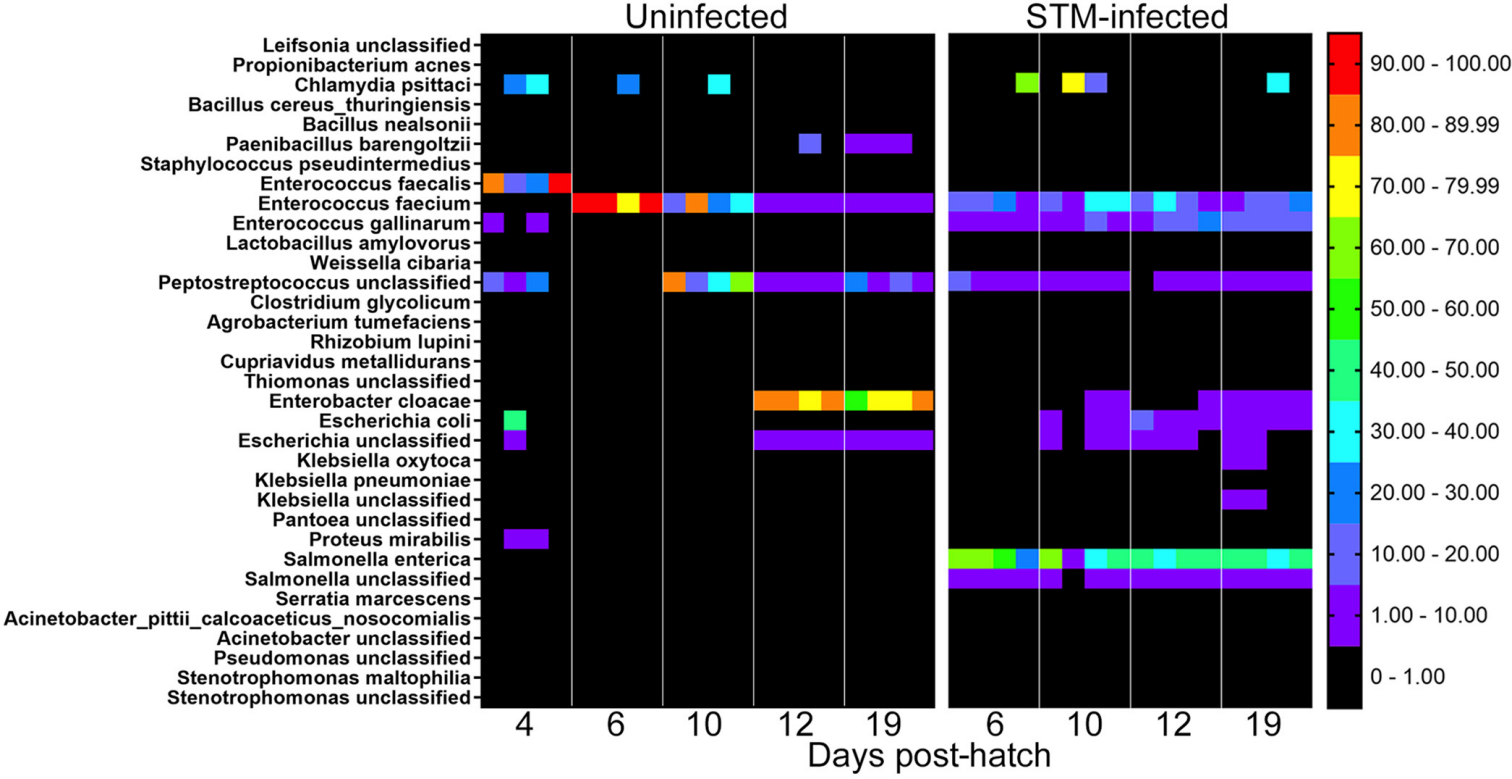
Taxonomic profile of cecal microbiota development based on shotgun metagenomic analysis. DNA extracted from the cecal contents of uninfected (left panel) or STm-infected (right panel) chicks was used for shotgun sequencing. Taxonomic profiling was analyzed using MetaPhlAn2 and StrainPhlAn2 tools.

In order to identify the species present in the cecal microbiota at different time points postinfection, we performed shotgun sequencing on cecal samples from uninfected and STm-infected chicks at days 4, 6, 10, 12, and 19 posthatch (Fig. 3). These experiments confirmed that the most prominent members of the microbiota in unperturbed chicks colonize the ceca in waves. In the first wave of cecal colonization at 4 days postinfection, Enterococcus faecalis (*Bacilli*) is the most highly abundant member of the cecal microbiota. By day 6 postinfection, these appear to be replaced by Enterococcus faecium (*Bacilli*), and replaced again by day 12 postinfection with Enterobacter cloacae (*Gammaproteobacteria*). In STm-infected chicks, this analysis identified the presence of *Bacilli* (*Enterococcus* spp.) and *Gammaproteobacteria* (Salmonella), as expected.

### Untargeted metabolomics analysis of cecal contents suggests the presence of common and unique metabolites.

**FIG 4:**
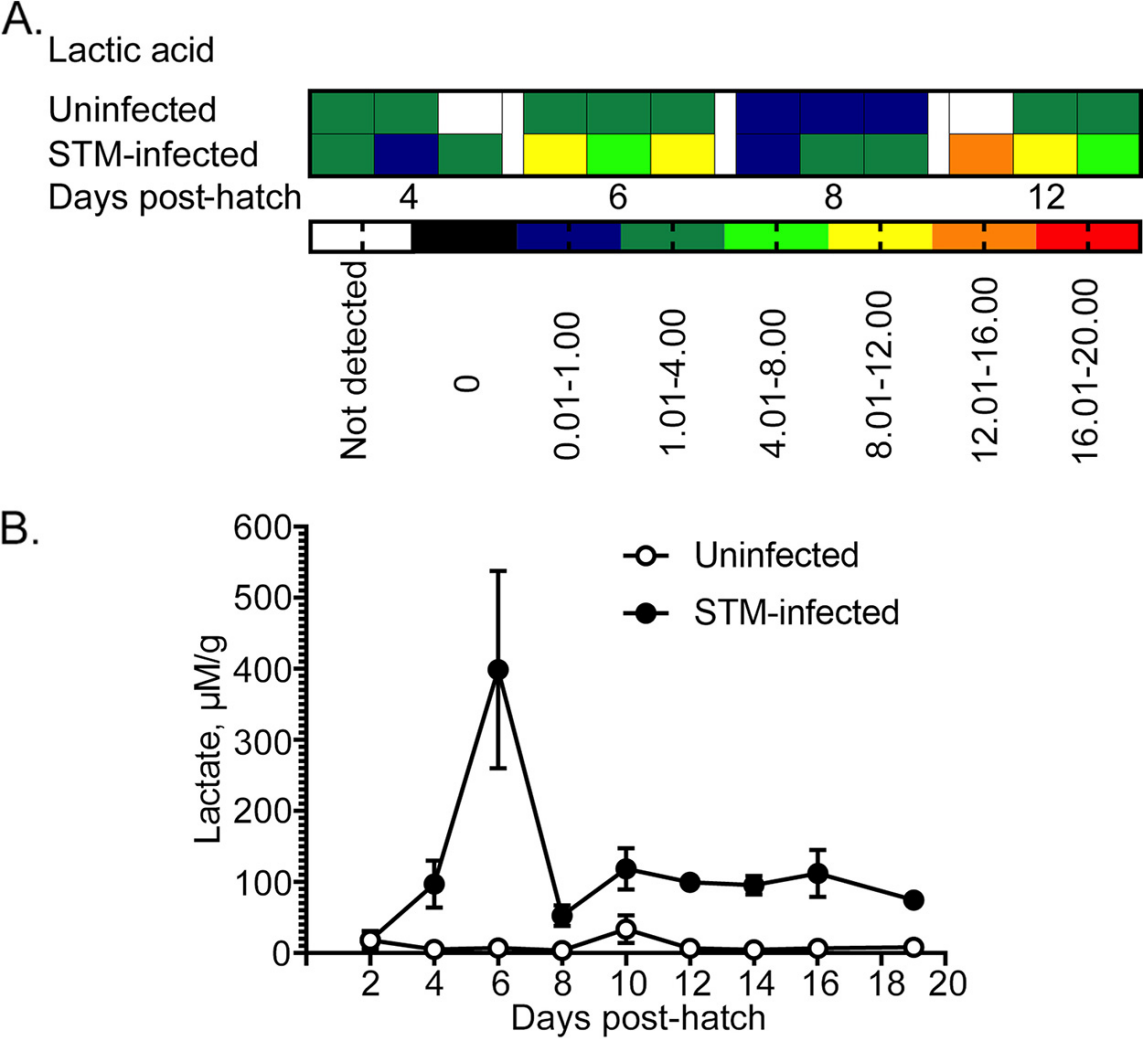
The presence of elevated levels of lactate in the ceca of Salmonella-infected chicks compared to uninfected birds was confirmed by GC-MS analysis. (A) A nontargeted metabolomic approach identified differences in lactic acid concentrations between infected and uninfected chicks (also Fig. S1, red box). (B) Quantification of lactate content in the cecal contents of uninfected (open circles) and STM-infected (black circles) chicks determined using GC-MS multiple reaction monitoring mode.

We performed an untargeted analysis of metabolites from the ceca of both groups of chick from days 4, 6, 8, and 12 posthatch (see Fig. S1 in the supplemental material) to understand the metabolic activities of both microbial communities. The results of this analysis showed differences in metabolic composition between the unperturbed and STm-infected groups (see Fig. S1). Lactic acid, for example, was detected in higher quantity in the ceca of infected chicks versus unperturbed chicks in this analysis (Fig. 4A). Using gas chromatography-mass spectrometry, we quantified the amount of lactate in the ceca of birds of both groups over 19 days of life (Fig. 4B). This analysis confirmed the metabolomics result that lactate is present in much higher amounts in the ceca of STm-infected chicks versus the unperturbed chicks. These data support our metagenomic result, suggesting that enterococci are an important and stable member of the cecal community in chicks after STm infection.

10.1128/mbio.02444-22.1 FIG S1 Nontargeted metabolomics analysis of the chicken cecal content. Cecal content was collected from uninfected (left panel) and STM-infected (right panel) chicks and analyzed by GC-MS. Download FIG S1, PDF file, 1.4 MB. Copyright © 2022 Bogomolnaya et al. 2022 Bogomolnaya et al. https://creativecommons.org/licenses/by/4.0/ This content is distributed under the terms of the Creative Commons Attribution 4.0 International license.

**FIG 5:**
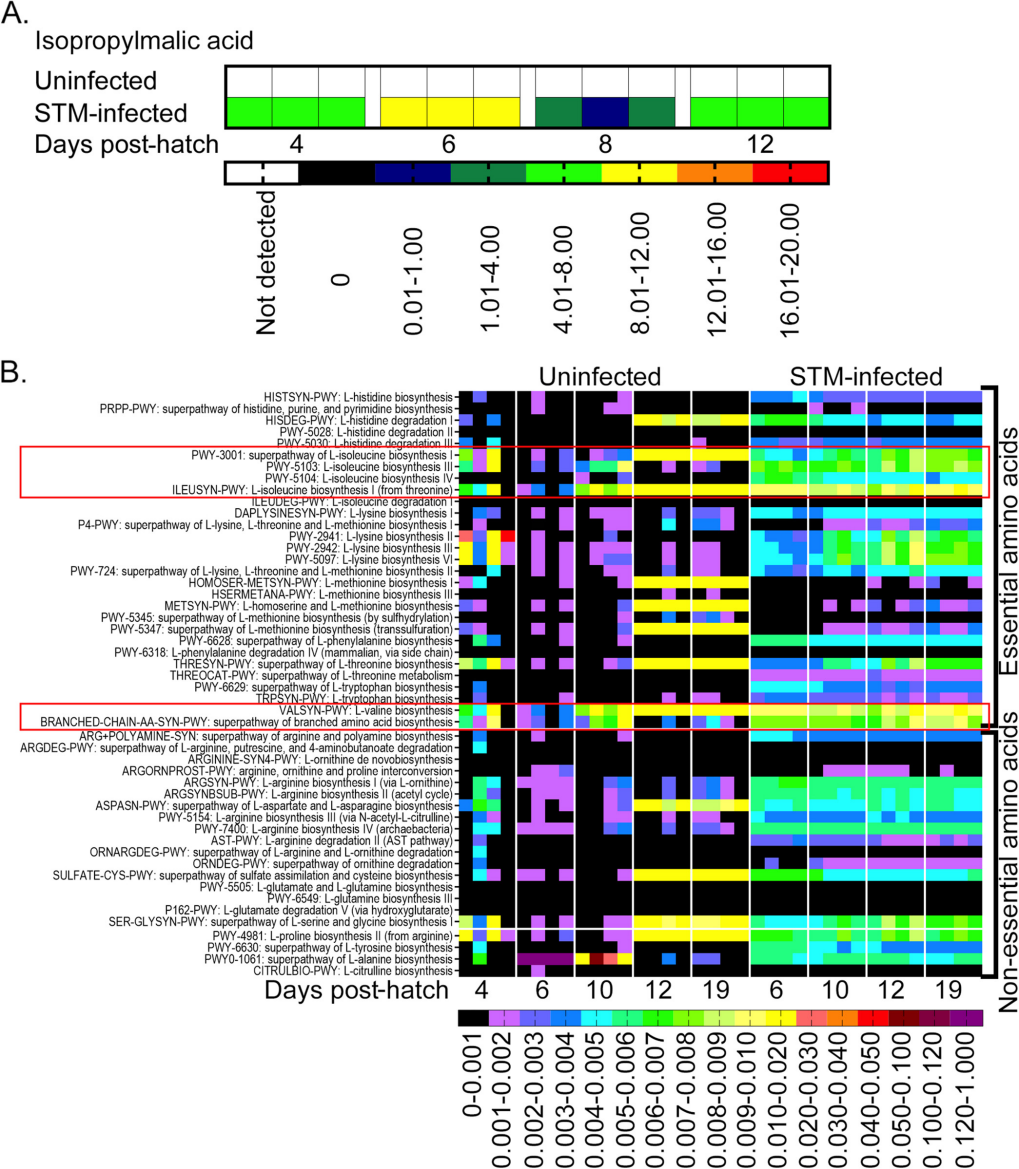
The presence of 2-isopropylmalic acid detected by nontargeted metabolomics and the higher abundance of genes involved in BCAA biosynthesis in the cecal contents of STm-infected chicks suggests the association of this pathway with STm colonization. (A) 2-isopropylmalic acid, an intermediate product in the biosynthesis of leucine, was detected by untargeted metabolomics only in the cecal samples collected from STm-infected chicks (also see Fig. S1, red box). (B) Functional profiling and relative abundance levels of the amino acid biosynthesis and degradation genes present in the ceca of uninfected (left panel) and STM-infected of infected chicks (right panel) was constructed using the HUMAnN2 tool.

**FIG 6:**
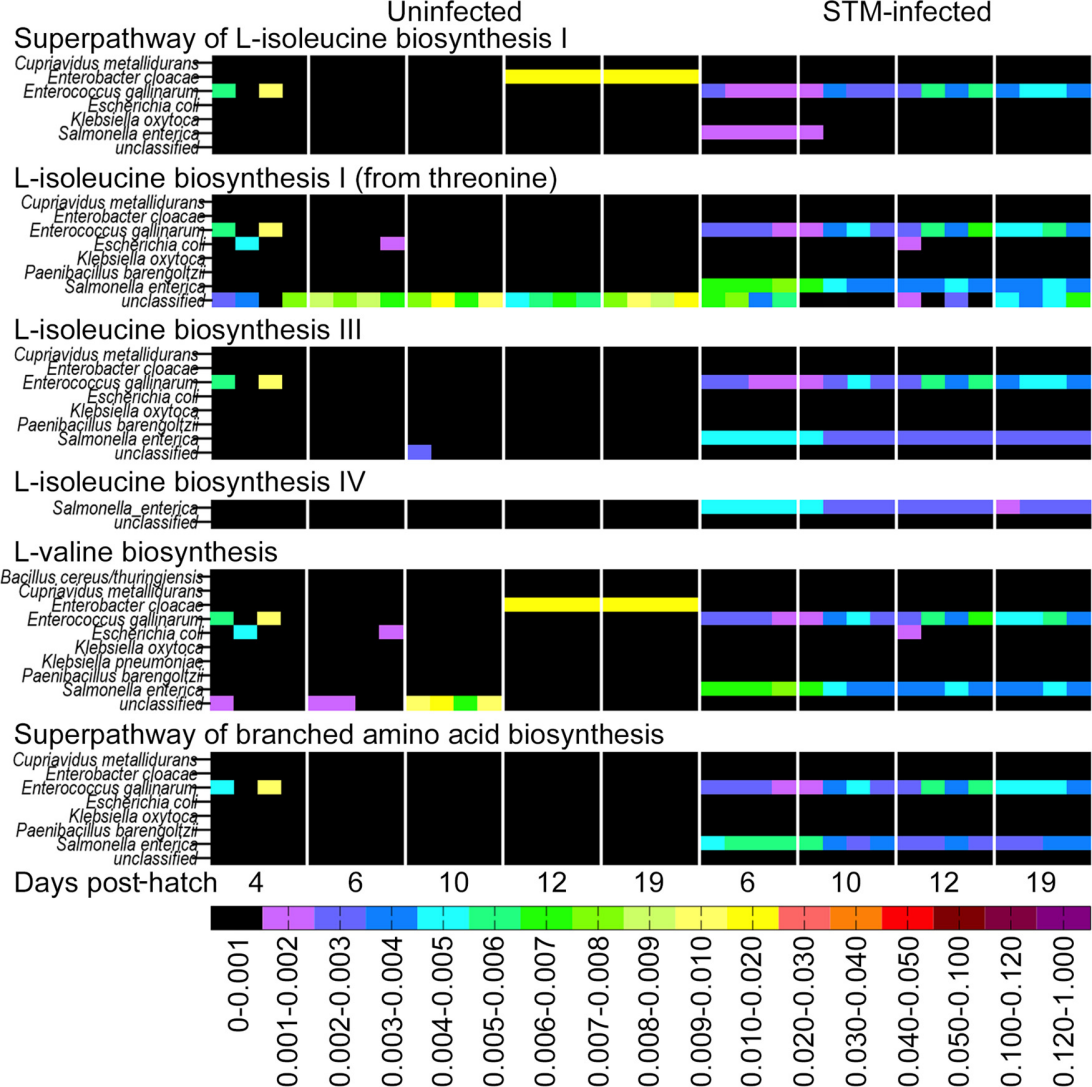
Functional profiling of the shotgun sequencing data indicates that most genes involved in branched amino acid synthetic pathways are from Salmonella enterica and Enterococcus gallinarum in the infected ceca. The analysis of amino acid biosynthesis and degradation genes present in the ceca of uninfected (left panel) and STM-infected (right panel) of infected chicks was performed using the HUMAnN2 tool.

In addition, metabolites that were present in one group of chicks only were revealed. One of these metabolites, 2-Isopropylmalic acid, was uniquely present in the ceca of chicks infected with STm (Fig. 5A; see also Fig. S1 in the supplemental material). 2-Isopropylmalic acid is an intermediate product in the biosynthesis of leucine from 2-ketovaline. Functional profiling of the shotgun sequencing data suggested that the microbial community from STm-infected chicks was characterized by a higher abundance of genes involved in branched-chain amino acid (BCAA) biosynthesis (Fig. 5B). Genes in pathways for the biosynthesis of the BCAAs isoleucine and valine were particularly abundant in the ceca of chicks from the day of infection until the termination of the experiment in these two groups, but not in unperturbed chicks. Detailed analysis of these data suggested that the majority of genes contributing to BCAA biosynthesis belonged to Salmonella enterica (Fig. 6). These data in combination with the presence of abundant 2-isopropylmalic acid in STm-infected chicks suggested that the biosynthesis of isoleucine and valine is particularly important for STm growth in the developing chick intestine.

### BCAA biosynthesis is critical to STm colonization in the ceca of developing chicks.

**FIG 7:**
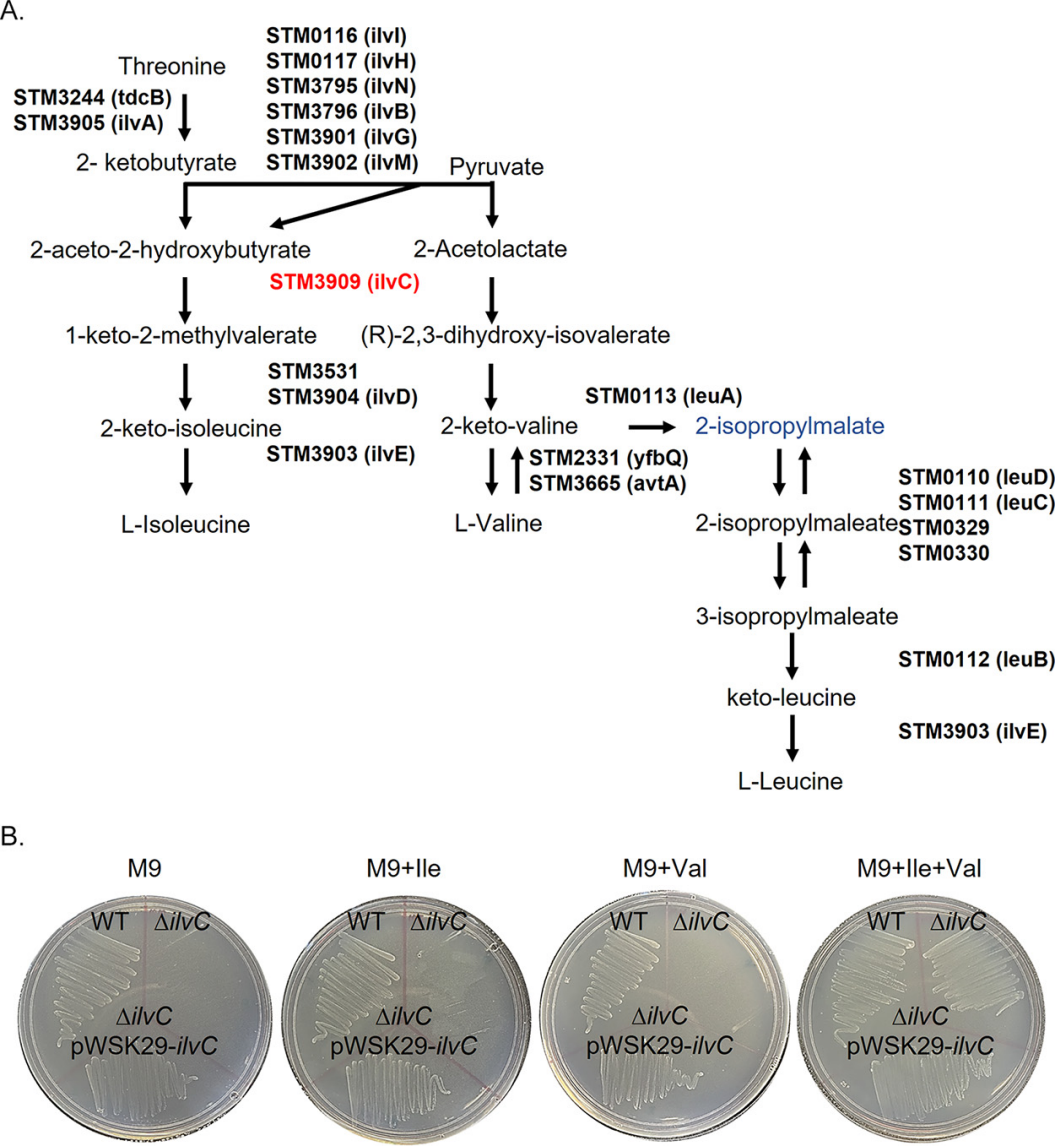
The *STM3909* (*ilvC*) gene is essential for BCAA synthesis in Salmonella Typhimurium. (A) An overview of BCAA synthesis in Salmonella Typhimurium. (B) The *STM3909* mutant requires the presence of l-isoleucine (Ile) and l-valine (Val) for growth in minimal medium. The strains shown are as follows: wild type, *ΔSTM3909* (*ΔilvC*), and *ΔSTM3909* bearing pWSK29::*STM3909*.

Both the biosynthesis of l-isoleucine from threonine and of valine from pyruvate require the conversion of (*S*)-2-acetolactate to (2*R*)-2/3-dihydroxy-3-methylbutanoate using the enzyme IlvC [(2*R*)-2,3-dihydroxy-3-methylbutanoate:NADP^+^ oxidoreductase] (Fig. 7A). We generated an *ilvC* deletion mutant (Δ*ilvC* or Δ*STM3909*) and tested the ability of this mutant to grow on both minimal media and minimal media supplemented with l-isoleucine, l-valine, or both. Mutants lacking *ilvC* failed to grow on minimal media or on minimal media supplemented with either l-isoleucine or l-valine (Fig. 7B). Supplementation of minimal media with both l-isoleucine and l-valine or the return of an intact *ilvC* gene in *trans* restored the ability of the *ilvC* mutant to grow. Thus, deletion of *ilvC* creates an l-isoleucine and l-valine auxotrophy that is reversible with the introduction of an intact copy of *ilvC* in *trans* (Fig. 7B).

**FIG 8:**
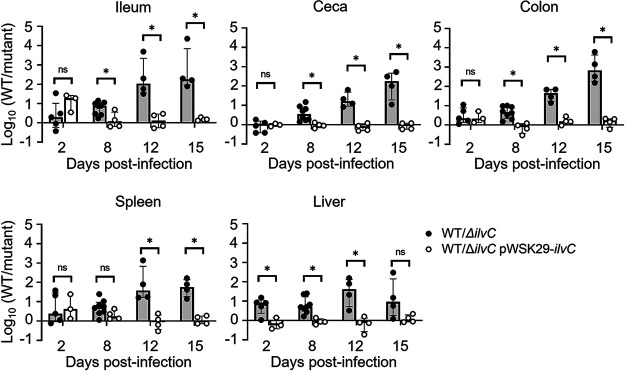
BCAA biosynthesis is required for Salmonella colonization of 4-day-old chicks. Chicks were infected with 10^8^ CFU of a 1:1 mixture of the *ΔilvC* mutant and wild-type strains (black circles) or of *ΔSTM3909/*pWSK29::*STM3909* mutant and wild-type strains (open circles). Chicks were euthanized on days 2, 8, 12, and 15 postinfection, and ilea, ceca, colons, spleens, and livers were collected for enumeration of the CFU. Statistical significance was determined using a Student *t* test. *, Significant difference (*P < *0.05) in the mutant/wild type ratio in the collected tissues compared to that of the inoculum.

We tested the ability of a deletion mutant in *ΔilvC* (*ΔSTM3909*) to colonize 4-day-old chicks using competitive infections with the otherwise isogenic wild type (Fig. 8). Although initial colonization between the deletion mutant and the wild type was equal, deletion of *ilvC* severely restricted the growth of the mutant in the intestinal tract and systemic sites in chicks during 15 days of infection. Returning an intact copy of *ilvC* in *trans* restored the colonizing ability of the *ilvC* deletion mutant (Fig. 8).

## DISCUSSION

In modern commercial poultry production, newly hatched chicks have no contact with adult birds, and the microbial communities present in the environment function as the inoculum that can shape the development of chicken gut microbiota. The process of intestinal colonization by bacterial organisms is rapid and very robust. Within 1 day of hatching, the bacterial densities in the ilea and ceca of broiler chickens reached 10^8^ and 10^10^ CFU/g of ingesta, respectively. The bacterial concentration further increases during first 3 days of the life of the chick reaches 10^9^ and 10^11^ CFU/g of ileal and cecal content and remains relatively stable for the following 30 days (20).

In agreement with previous reports (21–23), we found that during the first several days posthatch, the diversity of cecal microbiota is very low and composed primarily of *Proteobacteria*. Exposure of the chicken gut to *Enterobacteriaceae* at the day of hatch can induce mild, nearly undetectable inflammation leading to immune tolerance of related bacteria, including Salmonella (24–26). Accordingly, the ceca of chickens infected with STm are only mildly inflamed despite a high bacterial burden. Heavy intestinal colonization with Salmonella does not affect chicken weight gain, in agreement with previous work.

We confirmed that the gut microbiota of untreated chicks undergoes the expected dynamic reorganization during first 2 weeks of chick development (21–23, 27–29) and results in the replacement of the pioneer proteobacterial population by *Firmicutes*. Interestingly, *Bacteroidetes*, reported to be another common phylum in the chick ceca (30–32), was not abundant in the microbial community of the Leghorn chicks we used. Perhaps diet and geographic location, known to play a role in the modulation of chicken microbiota (33, 34), contributed to this difference.

Mammalian gut microbial communities provide the host with complementary diet-dependent metabolic potential to support the degradation of macromolecules associated with a typical diet. Thus, the gut microbiota of carnivores is enriched with genes needed for breakdown of proteins. In contrast, the microbial communities present in herbivores are enriched in genes involved in the biosynthesis of amino acids and in the degradation of complex polysaccharides (35). However, in birds the gut microbiota is shaped by a complex interaction between diet, evolutionary history, and genetic, ecological, behavioral, and environmental factors (36, 37). The avian gut microbiota is largely dominated by *Firmicutes* and *Proteobacteria* with a lower abundance of *Bacteroidetes* and *Actinobacteria* (37).

Introduction of STm to the developing microbial community results in a major shift in the phyla in the cecal population. We detected a noticeable increase in *Lactobacillales* abundance previously linked with the presence of Salmonella in the chicken gut (38). The presence of STm may create a microaerophilic environment in the gut lumen, thereby promoting the growth of lactic acid bacteria and limiting the expansion of *Clostridia*.

The gut microbiome as a complex ecosystem makes an important contribution to chicken metabolism by providing enzymes for the breakdown of macronutrients and the synthesis of vitamins. Although chickens can survive without ceca (39), this organ plays an important role in recycling of urea, water regulation, and carbohydrate fermentation (40–42). Well-functioning ceca cover approximately 10% of chicken energy needs (42). However, the full fermentation capacity of ceca in broilers is not reached before 28 days of age (43). Nontargeted analysis of cecal metabolites reflected the dynamic modulation of the microbiota. As expected (44), lactate, the main product of glucose fermentation by *Enterococcus* (45), was detected in the ceca of uninfected chicks at low levels. Accordingly, the increased presence of Enterococcus gallinarum in the guts of STm-infected chicks resulted in higher accumulation of this metabolite.

Our current understanding of metabolic changes caused by Salmonella colonization in the intestine is based on cecum metabolite profile alterations caused by Salmonella Enteritidis inoculation of 2-week-old UCD-003 layer chicks (46). Our metabolomic analysis showed a limited overlap with this published data (46). This discrepancy is likely due to the differences in Salmonella serotypes used, and to differences in the ages of the chicks at the time of infection. Nevertheless, at least two previously identified metabolites, 2-isopropylmalic acid and 4-hydroxybenzoic acid, were consistently more abundant in the ceca of chickens infected with either Salmonella Enteritidis (46), or Salmonella Typhimurium. 2-isopropylmalic acid is an intermediate product in the biosynthesis of leucine from 2-keto-valine. Our metagenomic analysis confirmed the increased abundance of BCAA biosynthetic genes in the ceca of STm-infected chicks mainly originating from two bacterial species: Salmonella enterica and Enterococcus gallinarum.

The BCAAs isoleucine, valine, and leucine play an important role in the growth, production performance, immunity, and intestinal health of chickens. Inclusion of BCAAs in the chicken diet is essential for the proper functioning of the immune system and the maintenance of intestinal mucosal integrity (47). However, our current understanding of the effect of BCAAs on bacterial diversity in the gut is very limited. BCAAs are quickly depleted from pig intestines by the microbial community (48). In turn, supplementation of protein-restricted piglet diet with BCAAs facilitated overgrowth of *Lactobacillales* (49). BCAA restrictions from the diet resulted in increased susceptibility of mice to Salmonella Typhimurium (50). Thus, BCAA biosynthesis plays an important role in Salmonella pathogenesis of orally infected mice (51). Our data demonstrate the importance of this biosynthetic pathway not only for the survival in mammalian host but also for successful Salmonella engraftment in the developing microbial community of young chicks.

Our multifaceted approach reveals the consequences on microbial community structure caused by Salmonella colonization, reveals the changes in metabolites present in the altered gut environment and illustrates the requirement for BCAA biosynthetic pathway for chicken gut colonization.

## MATERIALS AND METHODS

### Bacterial strains and media.

Bacterial strains used in this study are listed in Table 1. The Salmonella strains used in this study were derived from Salmonella enterica subsp*. enterica*, serovar Typhimurium ATCC 14028s (American Type Culture Collection, Manassas, VA). Strains were routinely grown in Luria-Bertani (LB) broth or M9 minimal medium with appropriate antibiotics. When strains were used for infection of chicks, overnight cultures were grown at 41°C with aeration and appropriate antibiotics. Antibiotics and amino acids were used at the following concentrations: 100 mg/mL nalidixic acid, 100 mg/mL chloramphenicol, 100 mg/mL carbenicillin, 500 mg/L l-isoleucine, and 500 mg/L l-valine.

**TABLE 1:** Strains used in this study

Strain	Description^*a*^	Source or reference
HA420	ATCC14028, spontaneously Nal^r^	60
HA1632	HA420 *ΔSTM3909*::Cm^r^	This study
HA1633	HA420 *ΔSTM3909*::Cm^r^/pWSK29-*STM3909*; Amp^r^	This study

a Cm^r^, chloramphenicol resistance; Amp^r^, ampicillin resistance; Nal^r^, nalidixic acid resistance.

A deletion mutant lacking *STM3909* (HA1632; *ΔSTM3909*), the mutant complemented with a wild-type copy of *STM3909* (HA1633; *ΔSTM3909* bearing pWSK29::*STM3909*), and the wild-type strain (WT; HA420) were grown in LB broth and in M9 minimal medium with or without amino acid supplementation, at 37°C with aeration, and plated on LB agar plates containing appropriate antibiotics.

### Plasmid construction.

A plasmid containing an intact *ilvC* gene (*STM3909*; 1,476 bp), with flanking regions located ~200 bp upstream the gene, was purchased from GenScript Biotech. In short, an oligonucleotide encompassing this region was synthesized, cut with KpnI and BamHI restriction enzymes, and ligated into pWSK29 cut with the same enzymes. This plasmid was transformed into HA1632 (*ΔSTM3909*) to generate the complemented mutant HA1633 (*ΔSTM3909*, pWSK29::*STM3909*).

### Ethics statement.

All animal experiments were conducted in compliance with the *Guide for the Care and Use of Laboratory Animals* of the National Institutes of Health. The protocol has been reviewed and approved by the Animal Care and Use Committee (IACUC) of Texas A&M University (AUP permit 2017-0137).

### Chick hatching.

SPF eggs (Charles River SPA-FAS) were incubated in an egg hatchery (GQF Manufacturing Co., Savannah, GA) at 38°C with 58 to 65% humidity during a 21-day hatching period. During the first 18 days, the eggs were turned two to three times a day and moved to a hatching tray during the last 3 days of the hatching period, where they remained still, to allow the chicks to hatch. Newly hatched chicks were moved to warmed brooders and maintained at 32 to 35°C. Birds had *ad libitum* access to water and irradiated antibiotic-free 3958 Teklad Laboratory Chick Diet (Envigo Teklad, Cambridgeshire, UK).

### Individual infection in chicks.

Seventy-seven chicks were hatched and either left unperturbed (control) or infected with STm HA420. A total of 38 chicks were infected with STm by gavage on day 4 posthatch with 10^8^ organisms. Inocula were serially diluted and plated for accurate determination of the concentration of organisms in the inoculum. Chicks in both groups were monitored daily for weight change and for signs of infection. At 2-day intervals beginning on day 2 and up to day 16 posthatch, four to five chicks from each group were euthanized, and the ileal, cecal, and colonic contents, as well as livers and spleens, were collected for CFU enumeration. A final group of chicks from each treatment group was euthanized at day 19 posthatch. The cecal contents were also used for microbiota analysis and metabolic profiling.

### Competitive infection in chicks.

SPF chicks (*n* = 36) were orally infected with an inoculum of 10^8^ CFU of a 1:1 mixture of either WT HA420 plus HA1632 (*ΔSTM3909*) or WT (HA420) plus HA1633 (*ΔSTM3909 pSTM3909*) on day 4 posthatch. After infection, the chicks were monitored twice daily for signs of disease. On days 2, 8, 12, and 15 postinfection (i.e., 6, 12, 16, and 19 days posthatch) four to eight chicks were humanely euthanized. The ceca, ilea, colons, spleens, and livers were excised, homogenized in phosphate-buffered saline (PBS), and serially diluted and plated on LB plates with appropriate antibiotics for CFU enumeration to calculate the competitive index between the two infecting strains.

### 16S rRNA gene sequencing.

Cecal contents collected from chicks at days 2, 4, 6, 8, 10, 12, 14, 16, and 19 posthatch were flash frozen in liquid nitrogen and stored at −80°C. Bacterial DNA was extracted from cecal content using a QIAamp PowerFecal DNA kit (Qiagen). The 16S rRNA gene V3-4 variable region was amplified with 515F (GTGYCAGCMGCCGCGGTAA) and 806R (GGACTACNVGGGTWTCTAAT) primers to construct Illumina compatible sequencing libraries. All libraries were purified using gel electrophoresis to remove background amplification and primer dimers. Libraries were sequenced to generate 150-bp paired-end reads using the Illumina MiSeq platform. The raw 16S rRNA gene sequence data were processed with QIIME 2 (Quantitative Insights into Microbial Ecology) software package (52). Operational taxonomic unit picking was performed using the Silva database (53).

### Shotgun metagenomic sequencing.

Bacterial DNA was extracted from the cecal contents collected at days 4, 6, 10, 12, and 19 posthatch from uninfected chicks and at days 6, 10, 12, and 19 posthatch from Salmonella-infected chicks, as described above, and quantified using a Qubit dsDNA fluorometric assay (Thermo Fisher). DNA quality was assessed by DNA ScreenTape assay using TapeStation 2200 (Agilent). Samples were normalized and used to generate sequencing libraries using the Nextera DNAflex Library preparation kit according to the manufacturer’s protocol (Illumina). Normalized libraries were pooled in an equimolar ratio and then sequenced on a single lane of a NovaSeq S4 2 × 150 flow cell (NovaSeq 6000; Illumina) with at least 50 million reads per sample.

### Data processing.

After sequencing, a total of ~2.3 billion paired-end 150-bp reads were generated across 36 samples. The reads were then processed using bioBakery whole-genome sequencing metagenomic pipeline (54). Raw reads were processed using Kneaddata (https://huttenhower.sph.harvard.edu/kneaddata/) to remove adapter sequences and host (Gallus gallus) reads. Specifically, the Kneaddata workflow included trimming of overrepresented reads with FastQC (https://qubeshub.org/resources/fastqc), followed by the removal of adapter sequences using Trimmomatic (55). The remaining reads were further filtered using the Bowtie 2 tool (56) to remove chicken-related host reads. Filtered reads were used as an input for taxonomic profiling and strain identification using MetaPhlAn2 (57) and StrainPhlAn2 (58), respectively. HUMAnN2 tool (59) was then used for functional profiling and to detect abundant pathways in the sample-specific pangenomes. Unmapped reads remaining after this step were used to perform a translated search against the Uniref90 database using DIAMOND. Collected results were used for quantification of the relative pathway abundances.

### GC-MS metabolic profiling.

Cecal contents of infected and uninfected birds were collected, resuspended in sterile PBS, weighed, and placed on ice. Samples were vortexed for 2 min and then separated by centrifugation at 6,000 × *g* for 15 min at 4°C. Supernatants were mixed with 5 μM deuterated lactate (sodium l-lactate-3,3,3,-d_3_, CDN Isotopes) as an internal standard, transferred to the new tubes, and dried using a SpeedVac concentrator. Samples were dissolved by addition of pyridine in 1:1 ratio, sonicated for 1 min, and incubated at 80°C for 20 min. Next, the derivatization reagent *N*-*tert*-butyldimethylsilyl-*N*-methyltrifluoroacetamide with 1% *t*-BDMCS (*tert*-butyldimethylchlorosilane; Cerilliant) was added, and samples were incubated at 80°C for 1 h. After centrifugation at 14,000 rpm for 1 min, derivatized samples were transferred to autosampler vials for gas chromatography-mass spectrometry (GC-MS) analysis (Shimadzu, TQ8040) using a Rtx-5Sil MS column (30 m × 0.25 mm × 0.25 μm; Shimadzu). The injection temperature was 250°C, and the injection split ratio was set to 1:100 with an injection volume of 1 μL. The oven temperature started at 50°C for 2 min, increasing to 100°C at 20°C per minute and to 330°C at 40°C per min, with a final hold at this temperature for 3 min. The flow rate of the helium carrier gas was kept constant at a linear velocity of 50 cm/s. The interface temperature was 300°C. The electron impact ion source temperature was 200°C, with a 70-V ionization voltage and a 150-μA current. For qualitative experiments, Q3 scans (range, 50 to 550 *m/z*; 1,000 *m/z* per s) were performed, and putative compounds were identified by searching the Shimadzu database.

To quantitatively measure lactate and deuterated lactate, multiple reaction monitoring was used with a target ion *m/z* 261 > 233 and a reference ion *m/z* 261 > 189 for lactate and a target ion *m/z* 264 > 236 and a reference ion *m/z* 264 > 189 for deuterated lactate, respectively.

### Data availability.

The data underlying this work can be accessed at the Texas Data Repository (https://doi.org/10.18738/T8/RZLB3I). Raw sequence data from 16S rRNA gene sequencing is available at accession number PRJNA906507. If you are unable to access the data at these sites, the authors will also make all underlying data available upon request.
