# Supplementary figures and images for: Taxonomic and Metagenomic Analyses Define the Development of the Microbiota in the Chick
Source: mBio. 2022 Dec 8;14(1):e02444-22. doi: 10.1128/mbio.02444-22 (PMC9973254; doi:10.1128/mbio.02444-22)

Figure S1.

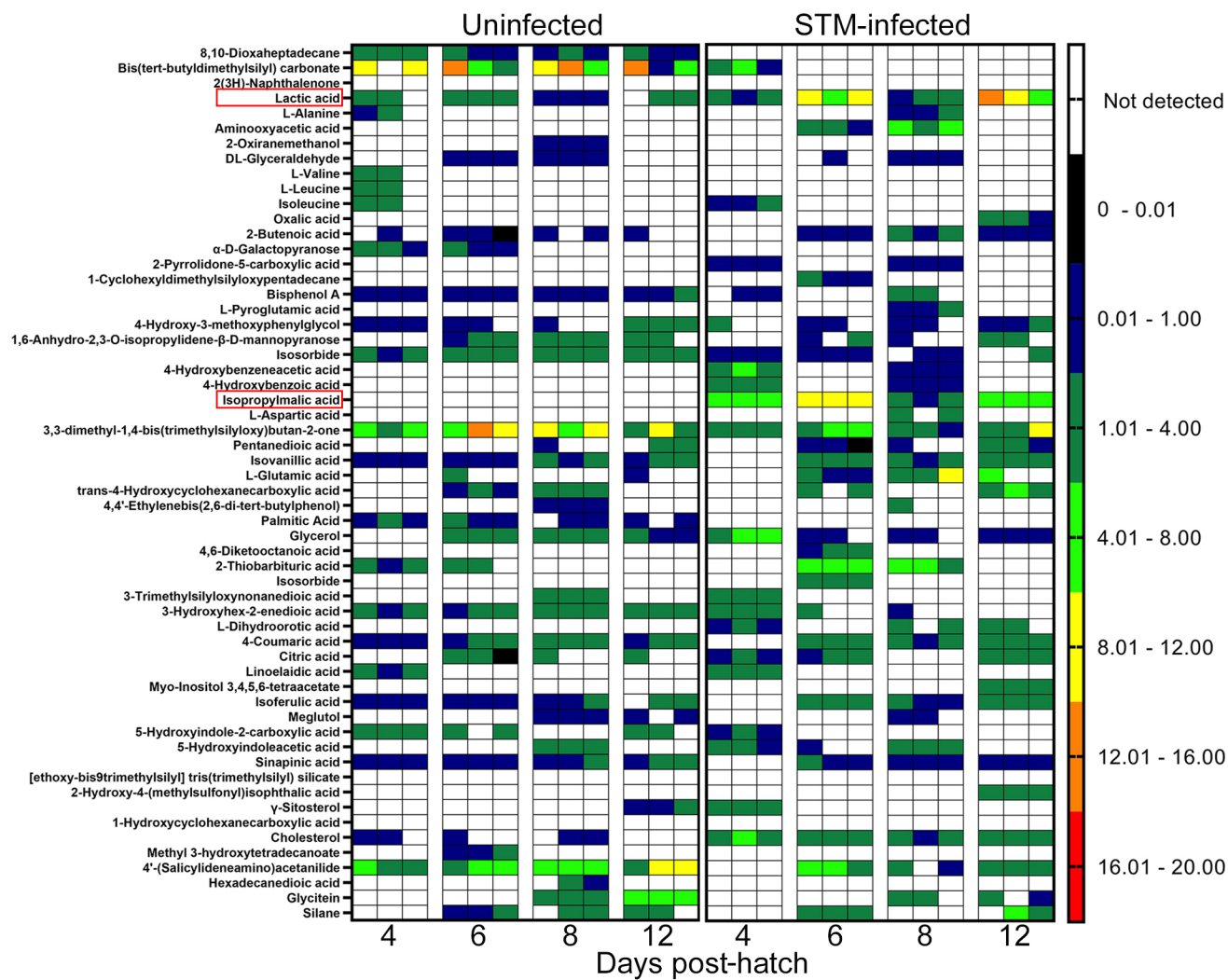

Supplement: FIG S1 [file mbio.02444-22-s0001.pdf]
